# The Tumorigenic Roles of the Cellular REDOX Regulatory Systems

**DOI:** 10.1155/2016/8413032

**Published:** 2015-11-22

**Authors:** Stéphanie Anaís Castaldo, Joana Raquel Freitas, Nadine Vasconcelos Conchinha, Patrícia Alexandra Madureira

**Affiliations:** Centre for Biomedical Research (CBMR), University of Algarve, Campus of Gambelas, Building 8, Room 2.22, 8055-139 Faro, Portugal

## Abstract

The cellular REDOX regulatory systems play a central role in maintaining REDOX homeostasis that is crucial for cell integrity, survival, and proliferation. To date, a substantial amount of data has demonstrated that cancer cells typically undergo increasing oxidative stress as the tumor develops, upregulating these important antioxidant systems in order to survive, proliferate, and metastasize under these extreme oxidative stress conditions. Since a large number of chemotherapeutic agents currently used in the clinic rely on the induction of ROS overload or change of ROS quality to kill the tumor, the cancer cell REDOX adaptation represents a significant obstacle to conventional chemotherapy. In this review we will first examine the different factors that contribute to the enhanced oxidative stress generally observed within the tumor microenvironment. We will then make a comprehensive assessment of the current literature regarding the main antioxidant proteins and systems that have been shown to be positively associated with tumor progression and chemoresistance. Finally we will make an analysis of commonly used chemotherapeutic drugs that induce ROS. The current knowledge of cancer cell REDOX adaptation raises the issue of developing novel and more effective therapies for these tumors that are usually resistant to conventional ROS inducing chemotherapy.

## 1. Introduction

**Figure 1:**
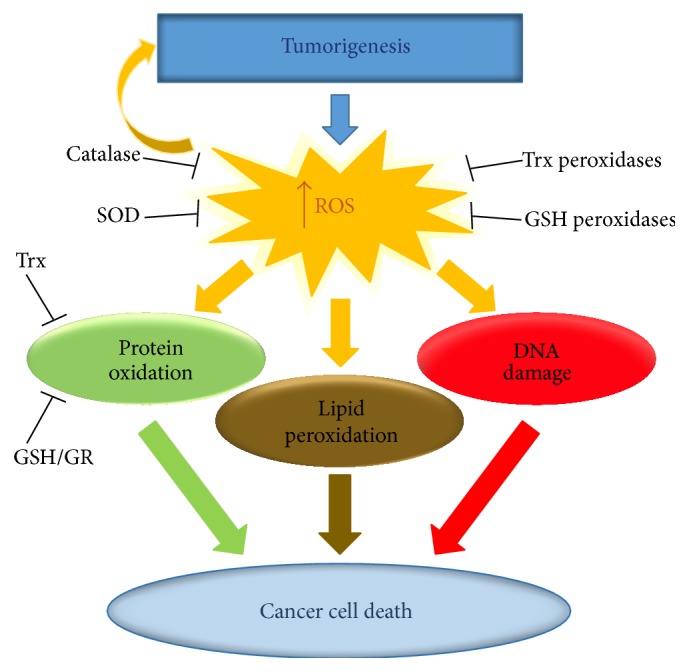
The cellular antioxidant systems. Tumor progression induces increasing oxidative stress. Cells have several antioxidant systems to directly inactivate ROS (e.g., Trx peroxidases, GSH peroxidases, catalase, and SOD) as well as REDOX regulatory systems that recycle/reactivate the ROS scavenging proteins and other REDOX sensitive proteins (e.g., PTPs, PTEN, and transcription factors).

Reactive oxygen species (ROS) are radical and nonradical oxygen-containing chemical molecules that show different degrees of reactivity. ROS include biologically important molecules such as superoxide anion (O_2_^•−^), hydroxyl radical (^•^OH) and hydrogen peroxide (H_2_O_2_). For a long time ROS have been tagged as harmful oxidants; however it is currently well established that the ROS molecule H_2_O_2_ constitutes an important second messenger in cell signaling transduction. This is due to its high diffusion rate across membranes particularly through the use of aquaporins and its ability to target reactive/REDOX sensitive cysteine residues in proteins [1–6]. Activation of NADPH oxidases (NOX) through the binding of growth factors, cytokines, neurotransmitters, and hormones to their receptors leads to the inducible production of O_2_^•−^ that is subsequently converted into H_2_O_2_, by the enzyme superoxide dismutase (SOD). To date, H_2_O_2_-dependent signaling has been shown to play a key role in the regulation of many cellular processes including differentiation, proliferation, migration, and apoptosis [3, 7–9]. Due to their reactive properties these species contribute, on different levels, to protein oxidation, lipid peroxidation, and/or DNA damage that can ultimately result in cell death or tumorigenesis (in case of DNA mutagenesis) [10, 11]. Consequently cells have several antioxidant systems to inactivate ROS and to recycle oxidized molecules; these include catalase, SOD, glutathione peroxidases, and thioredoxin peroxidases. The main cellular antioxidant systems responsible for recycling REDOX sensitive proteins are the thioredoxin (Trx) and glutathione (GSH) systems that reduce active cysteine residues present in ROS scavenging proteins (reestablishing their antioxidant function) and other proteins, whose functions are regulated by the oxidative status of key reactive cysteine residues (e.g., protein tyrosine phosphatases (PTPs), transcription factors, and the phosphatase and tensin homolog, PTEN) (Figure 1).

Several studies have shown an important role for ROS in tumor development [12, 13]. Cancer cells generally display elevated ROS compared to normal counterparts that give them a proliferative advantage and promote malignant progression [14]. In the tumor site the hypoxic cancer cells (in a low oxygen environment) typically show even higher levels of ROS compared to nonhypoxic cancer cells [15]. To deal with the increasing oxidative stress experienced as the tumor progresses, cancer cells upregulate the cellular antioxidant systems. This allows the cancer cells to maintain low to moderate levels of ROS (important for the activation of proliferative signaling pathways) while avoiding high levels of ROS that have damaging effects and can induce cell death [16–18].

A large number of currently used chemotherapeutic agents kill cancer cells in part through the generation of ROS. These drugs are less toxic to normal cells that have lower endogenous levels of ROS. Consequently, the upregulation of antioxidant proteins within the cancer cells will render them more resistant to chemotherapy [17–20].

In this review we will make a comprehensive examination of the current literature regarding the redox regulatory systems that become upregulated in cancer and their role in promoting tumor progression and resistance to chemotherapy. A better understanding of the molecular mechanisms used by cancer cells to adapt and survive to oxidative stress may also allow the development of more efficient chemotherapeutic treatments.

## 2. Sources of ROS in Cancer

The existing high levels of ROS typically observed in cancer cells are the result of accumulation of intrinsic and/or environmental factors.

### 2.1. Intrinsic Sources of ROS

**Figure 2:**
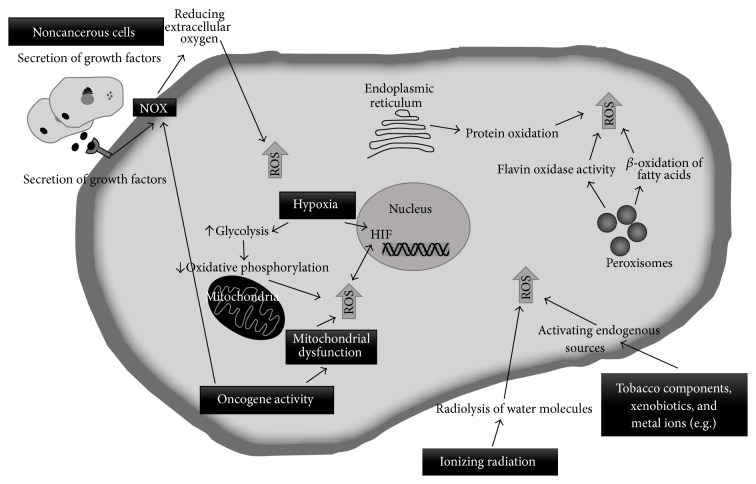
Sources of ROS in cancer. A number of intrinsic and extrinsic factors contribute to oxidative stress within the tumor as illustrated in the figure.

Several factors within the tumor site contribute to the generation of high levels of ROS in the cancer cells. The more relevant factors include hypoxia (low levels of oxygen), enhanced cellular metabolic activity, mitochondrial dysfunction, increased growth factor receptor mediated signaling transduction, oncogene activity, increased activity of oxidases, lipoxygenases, cyclooxygenases and thymidine phosphorylase, and the crosstalk between cancer cells and immune cells recruited to the tumor site (Figure 2) [21, 22].

Within the tumor mass, it has been well established that hypoxic cancer cells generally have higher levels of ROS compared to nonhypoxic counterparts. Hypoxia contributes to the production of ROS through different mechanisms. The major regulator of the hypoxic response is the transcription factor Hypoxia Inducible Factor (HIF). The regulation of the alpha subunit of this transcription factor (HIF-*α*) is dependent on intracellular levels of oxygen. HIF-*α* regulation is mediated by the action of prolyl hydroxylases (PHDs), enzymes that in the presence of normal concentrations of oxygen (normoxia) are able to hydroxylate HIF-*α* at two prolyl residues allowing the binding of the protein von Hippel-Lindau (pVHL). VHL in its turn recruits E3 ubiquitin ligases which target HIF-1*α* for proteasomal degradation [23–25]. Low levels of oxygen lead to the inhibition of PHDs and subsequent stabilization and accumulation of the HIF-*α* subunit. HIF-*α* translocates to the nucleus and binds to the HIF-1*β* subunit and cofactors such as CBP/p300 inducing the transcription of more than one hundred genes involved in promoting angiogenesis (formation of new blood vessels from preexisting blood vessels), glycolysis, epithelial-mesenchymal transition (EMT), proliferation, invasion, and recruitment of inflammatory cells to the tumor site [26, 27].

Hypoxia induced glycolysis and subsequent inhibition of oxidative phosphorylation at the mitochondrial membrane leads to an increase in ROS levels mediated by the mitochondrial complex III [28–30]. The hypoxic response also promotes the elevation of intracellular ROS via HIF dependent transcriptional activation of genes that encode for growth factors and their receptors. Binding of these growth factors to their receptors at the surface of cancer cells triggers signaling pathways that induce the activation of NADPH oxidases (NOX). NOX are responsible for the production of O_2_^•−^, which is then converted into H_2_O_2_.

HIF-1*α* and HIF-2*α* stabilization are also dependent on ROS. Gao et al. showed for the first time a decrease in tumor growth in mice treated with the antioxidant N-acetyl cysteine (NAC) that was traced to a redox mediated reduction in the levels of HIF [31]. In this way ROS is not only a by-product of hypoxia, but it also contributes to the hypoxic response by stabilizing its main regulator and thus creating a positive feedback loop. A number of studies have shown that the ROS produced in the mitochondria during hypoxia promote the oxidation/inactivation of prolyl hydroxylases (PHDs) thus stabilizing HIF-1*α* and HIF-2*α* [30, 32]. Furthermore the enhanced activation of NOX observed during hypoxia promotes NF-k*β* dependent* HIF-1α* transcription [33].

In cancer cells (as in normal cells), the mitochondria is thought to be the largest contributor to intracellular ROS that are generated as by-products of oxidative phosphorylation. The mitochondrial electron transport chain is composed of complexes I–IV and ATP synthase in the inner mitochondrial membrane. Complexes I and II oxidize NADH and FADH2, respectively, and transfer the resulting electrons to ubiquinol, which carries it to complex III. Within complex III the electrons are shuttled across the inner mitochondrial membrane to cytochrome c, which carries them to complex IV. The flow of electrons throughout the respiratory chain culminates at complex IV with the reduction of molecular oxygen to water. The incremental release of energy resulting from electron transfer is used to pump protons (hydrogen ions) into the intermembrane space generating a proton gradient, the dissipation of which is used by ATP synthase, to power the phosphorylation of ADP to ATP. Throughout this process, a small percentage of molecular oxygen can also undergo a one-electron reduction generating O_2_^•−^ [27, 34]. O_2_^•−^ is produced in complexes I and III of the electron transport chain and released to the intermembrane space (approximately 80%) or to the mitochondrial matrix (approximately 20%) [35, 36]. O_2_^•−^ can be converted into H_2_O_2_ that is able to cross the membranes and translocate to the cytoplasm and subsequently into the nucleus [1, 2]. In these different cellular compartments H_2_O_2_ can oxidize several cellular components, including proteins, lipids, and DNA (in the nucleus and mitochondria). Although peroxisomes are cellular organelles that are involved in ROS scavenging (via catalase mediated conversion of H_2_O_2_ into O_2_ and H_2_O) they also contribute to intracellular ROS production through the *β*-oxidation of fatty acids and by flavin oxidase activity [37]. The endoplasmic reticulum (ER) also contributes to the elevation of intracellular ROS levels via protein oxidation (disulphide bond formation) that occurs during the process of protein folding [38].

NOX constitute another important source of ROS in cancer cells. The NOX family consists of seven members, namely, NOX 1–5 and the dual oxidases DUOX 1-2. NOX are composed of transmembrane and cytoplasmic subunits that exert distinct functions (regulatory/catalytic). The catalytic subunit of all NOX isoforms contains six transmembrane domains in the N-terminal half, four conserved histidine residues located in the third and fifth transmembrane helices which coordinate two hemes, and a flavoprotein and an NADPH-binding domain in the cytosolic C-terminal region [39]. The NOX catalytic subunit transfers an electron from intracellular NADPH across the cytoplasmic membrane to oxygen via FAD and the two heme groups, with the second heme group being responsible for reducing extracellular molecular oxygen to produce O_2_^•−^. The O_2_^•−^ can be rapidly converted into H_2_O_2_ either spontaneously or through the action of SOD, which crosses the plasma membrane into the cytoplasm [39]. NOX are activated by various signaling proteins, including growth factors, cytokines, hormones, and neurotransmitters that are typically overexpressed within the tumor microenvironment leading to exacerbated activation of NOX and consequently increased levels of ROS within the cancer cells [3, 8].

Nowadays, it is well established that, in addition to the cancer cells, the tumor mass is constituted by innate immune cells (including macrophages, neutrophils, mast cells, myeloid-derived suppressor cells, dendritic cells, and natural killer cells); adaptive immune cells (T and B lymphocytes); and cells from the surrounding stroma that are recruited to the tumor site (consisting of fibroblasts, endothelial cells, and pericytes) [40]. These noncancerous cells constitute the tumor microenvironment and play a key role in tumorigenesis. Tumor associated cells are capable of inducing the generation of ROS in cancer cells through the secretion of various growth factors and other signaling proteins leading to the activation of NOX in the cancer cell. Neutrophils and macrophages can produce a rapid burst of O_2_^•−^ (primarily mediated via NOX) leading to the subsequent generation of H_2_O_2_ that can diffuse through the cytoplasmic membrane and into the neighboring cancer cells [35, 41]. Tumor associated macrophages (TAMs) can also produce nitric oxide within the tumor site through the activation of nitric oxide synthase 2 (NOX2). Nitric oxide reacts with superoxide to produce peroxynitrite (ONOO-) that can either be protonated to peroxynitrous acid and then spontaneously decompose into nitrogen dioxide (NO_2_) and ^•^OH (highly reactive/oxidizing) or react with carbon dioxide (CO_2_), yielding nitrosoperoxy carboxylate (ONOOCO_2_) which decomposes into carbonate radicals (CO_3_^•−^) and NO_2_ [42, 43].

### 2.2. Environmental Sources of ROS

Environmental sources of ROS can also significantly contribute to tumorigenesis. Ionizing radiation is the most studied source of environmental ROS and has been shown to play a role in all stages of carcinogenesis from cancer initiation to tumor progression. Ionizing radiation generates ROS through the radiolysis of water molecules and also by secondary reactions that can persist and diffuse within the cells [44].

Other environmental factors such as tobacco components, xenobiotics, chlorinated compounds, metal ions, barbiturates, and phorbol esters can generate ROS in the cells by metabolism to primary radical intermediates or by activating endogenous sources of ROS (Figure 2) [45].

## 3. Cancer REDOX Adaptation

**Figure 3:**
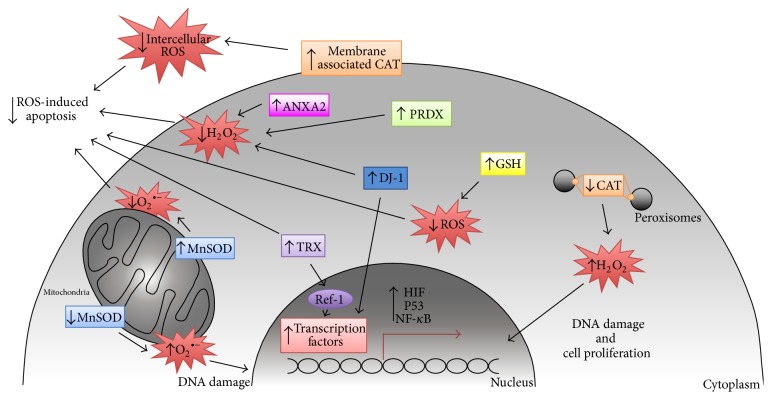
Antioxidant systems in cancer. Cancer cells undergo REDOX adaptation to survive and proliferate in an environment with increasing oxidative stress. Regulation of ROS levels by the cellular antioxidant systems is crucial to maintain a proliferative and mutagenic phenotype (associated with low/moderate levels of ROS) and avoid apoptosis or senescence (associated with high levels of ROS).

To balance the beneficial effects of low to moderate levels of ROS, which induce proliferative signaling pathways, and avoid the harmful oxidant effects of high levels of ROS that can damage proteins, lipids, and DNA, cancer cells undergo REDOX adaptation. Increasing evidence has demonstrated a crucial role for the cellular antioxidant systems in supporting tumor initiation, progression, and chemoresistance. Bellow we summarize what is currently known regarding the main antioxidant proteins/systems involved in cancer (Figure 3).

### 3.1. GSH System

The GSH and Trx systems are the main antioxidant systems responsible for the reduction of redox sensitive proteins in the cells.

The tripeptide GSH is the most abundant nonenzymatic antioxidant in the cell. GSH is a multifunctional antioxidant being able to (i) function as a cofactor of several oxidative stress detoxifying enzymes, for example, glutathione peroxidase (GPx), glutathione transferase, and others; (ii) directly scavenge ROS, such as hydroxyl radical and singlet oxygen; (iii) detoxify hydrogen peroxide and lipid peroxides through the catalytic activity of glutathione peroxidase; (iv) regenerate/reduce vitamins C and E; and (v) react with oxidized sulphenic acid and thiyl radical groups in proteins to form S-glutathionylated proteins (protein-SSG), protecting these proteins from further oxidation. S-Glutathionylated proteins can then be further reduced by the glutathione cycle through the action of glutathione reductase and small proteins such as glutaredoxin and thioredoxin [46–48].

It is well known that GSH metabolism is modified in a vast number of cancers. High levels of GSH have been reported in many types of cancer including breast, melanoma, and liver. Furthermore a direct correlation between GSH concentration and cellular proliferation and metastasis has also been established [18, 47, 49]. It is important to keep in mind that the GSH system has many components and that a slight alteration in one of them may have an instant result, disrupting the balance of cellular antioxidant response and REDOX homeostasis. Various modifications within the GSH system have been observed in cancer: higher levels of GSH-related enzymes, such as *γ*-glutamylcysteine ligase (GCL), *γ*-glutamyl-transpeptidase (GGT), and glutathione-S-transferases (GST), have been reported, as well as higher expression of GSH-transporting pumps [50–52]. Decreased GSH/GSSG ratio has also been found in advanced cancer patients; this can be explained by an increased generation of H_2_O_2_ that readily oxidizes GSH, turning it into GSSG [48].

The GSH system is a major contributor to chemotherapy resistance. This system participates not only in the cellular antioxidant defense, but also in drug-resistance metabolic processes including the detoxification and efflux of xenobiotics. The GSH system also participates in DNA repair processes as well as in the inactivation of the cancer cell proapoptotic pathway [18, 51, 53, 54]. GSH is able to directly interact with cisplatin and trisenox, inactivating these chemotherapeutics [55, 56]. For all these reasons, significant effort has been engaged at depleting cellular GSH levels to sensitize tumors to the cytotoxic effects of chemotherapeutic drugs.

Recently, a study showed that GSH plays a crucial role in tumor initiation [57]. Using the oncogene-induced murine model of mammary cancer (MMTV-PyMT) it was shown that a 75% depletion in the levels of GSH in these mice led to the formation of fewer tumors that progressed more slowly than those in mice with normal GSH levels [57].

### 3.2. TRX System

Thioredoxins (Trx) are small proteins (with a molecular weight of approximately 12 KDa) that repair oxidized proteins. Trx possess a conserved Cys-Gly-Pro-Cys redox catalytic site that is able to reduce oxidized thiols (reactive cysteine residues) in target proteins. Trx reductases (TrxR) recycle the oxidized Trx through the transfer of reducing equivalents from NADPH to the oxidized catalytic site of Trx (Trx-S_2_) reducing it to a dithiol (Trx-(SH)_2_) [9, 58]. Amongst all members of the thioredoxin system, Trx1 and TrxR1 have emerged as critical redox regulators and as potential therapeutic targets for many types of human cancers [59].

Trx are major protein disulphide reductases in the cell, being able to catalyze the reduction of disulphide bonds in proteins orders of magnitude faster than GSH [60]. Trx are involved in multiple redox-dependent signaling pathways in cancer by regulating redox-sensitive transcription factors (e.g., activator protein 1 (AP-1), NF-*κ*B, and p53); signaling proteins (e.g., protein tyrosine phosphatases, PTPs, and PTEN); and other antioxidant proteins involved in the regulation of these signaling cascades (e.g., peroxiredoxins). Reduced Trx has been shown to bind to and inhibit apoptosis signal-regulated kinase 1 (ASK1) [61]. ASK1 activates the c-Jun N-terminal kinase (JNK) and p38 mitogen-activated protein kinase pathways leading to apoptosis [62, 63]. Furthermore, Trx also play an important role controlling cancer cell growth through the supply of reducing equivalents for DNA synthesis [64].

Elevated levels of Trx have been reported in multiple types of cancers including cervical, liver, gastric, lung, and colorectal cancers [48, 64]. Many studies have also revealed a positive correlation between elevated levels of Trx in human tumors and resistance to chemotherapy [65–67]. Although a large number of reports have established a key role for the Trx system in cancer, a recent study seems to indicate that the Trx system is essential for tumor progression, but not initiation, where the GSH system plays a more prominent role [57].

### 3.3. SOD

Superoxide dismutase (SOD) is responsible for the dismutation of O_2_^•−^ into oxygen (O_2_) and H_2_O_2_ (that is less reactive than O_2_^•−^), thus providing an antioxidant defense [47]. In humans there are three isoforms of SOD: cytosolic Cu, Zn-SOD, mitochondrial Mn-SOD, and extracellular SOD (EC-SOD) [68]. Of these isoforms, the mitochondrial Mn-SOD has been more associated with cancer by functioning as a mitochondrial ROS switch.

A large number of reports demonstrated the elevation of Mn-SOD in tumors (e.g., lung, esophageal, gastric, head and neck, prostate, and colon) compared to matched normal tissues and elucidated that the enhanced expression of Mn-SOD resulted in increased cancer cell invasiveness, growth, survival, and metastatic behavior [69–75]. Overexpression of Mn-SOD is likely a compensatory mechanism to intrinsic ROS stress, especially in cancer cells with mitochondrial dysfunction and consequently increased levels of O_2_^•−^. In this situation enhanced expression of Mn-SOD might help the cancer cell to counteract superoxide stress and thus promote tumor progression. A potential mechanism for Mn-SOD induced metastasis is the H_2_O_2_-dependent increase in matrix metalloproteases expression [76, 77]. Interestingly, other reports demonstrated low activity of Mn-SOD in a wide range of cancers including cervical, breast, prostate, lung, and liver [78–82]. These studies also showed that overexpression of Mn-SOD in cancer cells led to decreased proliferation, anchorage-dependent growth, and invasiveness. However, these tumor suppressive properties of Mn-SOD were mainly observed* in vitro*, in experimental systems where cancer cells were artificial transfected with Mn-SOD-expressing vectors to induce high expression of this enzyme [78, 83]. Under these conditions, the cancer cell redox homeostasis can be significantly disturbed, leading to inhibition of cancer cell proliferation. However, the physiological relevance of such tumor-suppression function of Mn-SOD is still unclear in cancer cells* in vivo*. Also, Mn-SOD cancer cell growth suppression can be modulated by diminishing the levels of carcinogen-inducing O_2_^•−^ [84–86] and sensitization of cancer cells to cell death induced by different ROS-generating agents [87]. This dual effect of SOD acting as either a tumor promoter or a tumor suppressor should be addressed taking into account the nature of the ROS molecules generated in low or high SOD expressing cancer cells. O_2_^•−^ (which is elevated in tumors with low SOD activity) is a reductant of iron, which subsequently generates ^•^OH by transferring the electron to H_2_O_2_ [88]. The highly reactive ^•^OH can lead to enhanced mutagenesis via DNA oxidation. On the other hand, increased SOD activity will lead to enhanced levels of H_2_O_2_ that oxidizes reactive cysteine residues in proteins with high specificity, constituting an important second messenger in a wide variety of signaling pathways that activate proliferation, invasiveness, and metastasis. Furthermore, increased levels of O_2_^•−^ (associated with low levels of SOD) might be more beneficial to cancer cells in the early stages of tumor development where oxidative stress within the tumor mass is still low; increased levels of H_2_O_2_ (associated with high levels of SOD) might have a more prominent role at later stages of tumor progression, lowering unspecific damage of cellular components, while increasing H_2_O_2_ dependent signaling pathways. However there is still a lot of work to be done to investigate these hypotheses.

### 3.4. Catalase

Catalase (CAT) was originally regarded as a monofunctional peroxisomal enzyme that efficiently catalyzes the conversion of H_2_O_2_ into water and O_2_. This notion has evolved over the years and catalase is now considered a multifunctional enzyme that exhibits not only classic catalase activity, but also peroxidase [89, 90] and oxidase functions [91]. Moreover, catalase activity is not limited to the degradation of H_2_O_2_, as this enzyme also degrades peroxynitrite in an enzymatic reaction that, similarly to its classical reaction with H_2_O_2_, involves the formation of compound I (CATFe^IV^=O^•+^) [92, 93]. In addition, compound I of catalase can oxidize NO (CATFe^IV^=O^•+^ + 2^•^NO + H_2_O → CATFe^III^ + 2H^+^ + 2NO_2_^−^) [94, 95], whereas native ferricatalase (CATFe^III^) is reversibly inhibited by NO [96]. Thus, catalase has the potential to execute a central modulatory function at the cross point between H_2_O_2_ and NO/peroxynitrite-mediated signaling pathways.

Decreased CAT activity has been reported in cancer [69]. This low CAT activity leads to high levels of H_2_O_2_ and creates an intracellular environment favorable to DNA mutagenesis and to the activation of H_2_O_2_-dependent signaling pathways that typically trigger cell proliferation, invasion and metastatic phenotypes, consequently promoting tumor progression [35, 97, 98].

However, malignant cells may acquire membrane-associated catalase. Cancer cells exhibit enhanced NOX1-dependent superoxide anion generation [93, 99–102]. Various studies have shown that the overexpression of membrane-associated catalase on the outer surface of tumor cells has a protective role against apoptosis induced intercellular ROS signaling [93, 101–103]. Catalase-mediated protection from intercellular ROS signaling was found in all human cancer cell lines studied so far (more than 70 cell lines) [104], indicating that this might be an important mechanism for tumor survival. Efficient protection of tumor cells by membrane-associated catalase is not in contradiction to the finding that, in general, tumor cells have less catalase than normal tissue, as the surface of the tumor cell with its high local concentration of catalase represents a small proportion of the total cellular mass [105].

### 3.5. Peroxiredoxins

Peroxiredoxins (Prdx) are thioredoxin peroxidases that are divided into three classes: typical 2-cysteine peroxiredoxins (PrxI–IV), atypical 2-cysteine peroxiredoxins (PrxV), and 1-cysteine peroxiredoxins (PrxVI) [50]. Although their individual roles in cellular redox regulation and antioxidant protection are distinct, they all catalyze the reduction of H_2_O_2_, organic hydroperoxides, and peroxynitrite, to balance intracellular ROS levels [106, 107]. Due to their widespread distribution within the cell, Prdx are thought to constitute broad-range cellular antioxidant defenders. However, recent studies have demonstrated that typical 2-cysteine peroxiredoxins (Prdx I–IV) play key cell signaling regulatory roles by either interacting directly with specific REDOX-sensitive signaling protein(s) or by being located in the vicinity of REDOX-dependent signaling cascades (lipid rafts) and buffering ROS generated by NOX [108]. The 2-Cys Prx have been implicated in the regulation of diverse cellular processes, including proliferation, migration, apoptosis, and metabolism [108].

Overexpression of all known Prdx has been reported in many types of cancers including prostate, breast, lung, thyroid, and mesothelioma and is associated with cancer cell survival and tumor progression [109–112]. The ROS buffering and signaling regulatory (prosurvival) functions of Prdx have been suggested to play a role in tumor chemoresistance [106, 107, 112–114].

### 3.6. APE1/Ref-1

The Apurinic/apyrimidinic endonuclease/redox factor-1 (APE1/Ref-1) is a key enzyme that in addition to its DNA base excision repair function it also exerts important cellular functions in the REDOX control of multiple transcription factors involved in cancer progression, including NF-*κ*B, STAT3, AP-1, HIF-1, and p53 [115–118]. All of these transcription factors possess reactive cysteine residue(s) within their DNA binding domain that can be readily oxidized/inactivated by cellular ROS. APE1/Ref-1 is able to reduce these cysteine residue(s) through the exchange of a proton from one or two of its redox cysteine residues (Cys65, Cys93, Cys99, or Cys138). This restores the DNA binding capability of the transcription factors and subsequently promotes transcription of their target genes. Oxidized APE1/Ref-1 is then recycled/reduced by the Trx system [119].

APE1/Ref-1 is highly expressed in many cancers (e.g., breast, lung, liver, and gliomas) [120–122]. APE1/Ref-1 role in cancer progression is likely due to its ability to increase DNA repair and to activate antiapoptotic, inflammatory, and growth-promoting transcription factors [121]. APE1/Ref-1 has also been shown to be associated with tumor resistance to both ionizing radiation and chemotherapy [123–125]. Consequently, APE/Ref-1 has emerged as a promising therapeutic target for cancer treatment [115, 120, 126].

### 3.7. Transcription Factors

Transcription factors can indirectly counteract ROS in cancer. The transcription factor nuclear factor erythroid 2-related factor 2 (NRF2) binds to antioxidant response elements (ARE) in the regulatory regions of target genes and induces the transcription/expression of antioxidant enzymes [e.g., NAD(P)H:quinone oxidoreductase 1 (NQO1); SOD; catalase; heme oxygenase-1 (HO-1), TrxR, GST, GSH-synthetizing enzymes, and UDP-glucuronosyltransferases (UGTs)] [127–129]. NRF2 is considered to be the master regulator of intracellular antioxidant responses. NRF2 is regulated by its binding partner KEAP1 that targets NRF2 to proteasomal degradation [130]. It has been shown that hyperactive and oncogenic phosphoinositide 3-kinase- (PI3K-)AKT signaling which is commonly observed in a large number of cancer cells activate NRF2 [46]. NRF2 mutations have also been found in several types of tumors such as skin, esophageal, lung, ovarian, and breast cancers [131]. Generally these mutations are observed in the KEAP1-binding domain of NRF2 preventing KEAP1-mediated degradation of this transcription factor [131, 132]. Inactivating mutations in KEAP1 have also been identified [133]. All of these mutations lead to the constitutive stabilization of the NRF2 protein in the nucleus, providing strong evidence for a role for NRF2 in tumorigenesis. High expression of antioxidant NRF2 target genes has been linked to chemoresistance. For instance, HO-1 seems to be an important effector of NRF-2 induced chemoresistance in myeloid leukemias and inhibition of either NRF2 or HO-1 sensitizes the tumor to therapy [127]. It was also described that NRF2 enhances the cellular antioxidant capacity to counteract ROS mediated stress in cancer cells overexpressing oncogenic KRas [20].

Forkhead box O (FOXO) transcription factors belong to the large group of forkhead transcription factors that are all characterized by a conserved DNA binding domain termed the forkhead box [134]. FOXO activate the transcription of genes that encode for antioxidant proteins, including Mn-SOD, catalase, and sestrin 3 [135]. Although FOXO is generally considered to be a tumor suppressor and FOXO inactivation has been reported in a number of cancers [136, 137], recent studies have indicated that these transcription factors might also have protumorigenic functions. It has been shown that activated FOXO transcription factors support the survival of Acute Myeloid Leukemia (AML) cells [138]. FOXO transcription factors are inactivated by the PI3K-AKT pathway. Phosphorylation of FOXO by AKT leads to FOXO translocation from the nucleus into the cytoplasm, where they cannot exert their transcriptional function [134]. Interestingly, it has been observed that the FOXO genes are involved in chromosomal translocations that lead to alveolar rhabdomyosarcoma and acute lymphoblastic leukemia (ALL) [139]. Specifically, the paired box 3- (PAX3-) FOXO1 translocation is found in approximately 60% of all alveolar rhabdomyosarcoma tumors [140, 141]. The PAX3-FOXO1 fusion protein is insensitive to AKT inactivation, meaning that AKT cannot inhibit PAX-FOXO1 translocation to the nucleus or regulate its transcriptional activity [142]. However the contribution of FOXO transcriptional activity within the fusion protein is still fairly unknown and increased activity of PAX3 has been reported [141].

### 3.8. Dietary Antioxidant Compounds

Dietary antioxidants are nonenzymatic compounds that, although less specific compared to enzymatic antioxidants, appear to be important in cellular responses to oxidative stress including during tumorigenesis. For instance, vitamin C (ascorbic acid) is a water-soluble antioxidant which is mostly present in the cell in its reduced form, ascorbate, which acts as a reductant and enzyme cofactor and directly reacts with O_2_^•−^, ^•^OH, and various lipid hydroperoxides. Vitamin E (*α*-tocopherol) is a fat-soluble antioxidant, which acts as a free radical scavenger by converting free radicals into tocopheryl radicals, thus lowering their radical damaging abilities. Selenium is a nonmetal element that forms part of antioxidant selenoproteins such as glutathione peroxidase and thioredoxin reductase. Vitamin A (*β*-carotene) is a known fat-soluble antioxidant and its antioxidant property derives from its ability to quench singlet oxygen and trap peroxyl radicals. Deficiency of vitamin A causes oxidative stress, inhibiting cell repair function and causing cell damage [46, 47].

Much debate has focused on the consumption of antioxidant supplements by cancer patients undergoing chemotherapy due to concerns that the antioxidants may interfere with the mechanism of action of ROS generating therapeutic agents and subsequently decrease their efficacy [143, 144]. In this way, antioxidant supplementation might help the cancer cells to achieve redox homeostasis during tumor progression (generally accompanied by increasing oxidative stress). A recently published research using B-Raf and K-Ras induced lung cancer mouse models has demonstrated that the dietary antioxidant compound, vitamin E, can actually promote tumor progression [145]. This study further showed that vitamin E increased cell proliferation by decreasing ROS, DNA damage, and p53 expression in both mouse and human lung cancer cells [145].

Interestingly vitamin C has also been shown to have a prooxidant function. Vitamin C can reduce catalytic metals within the cells that in turn react with oxygen, producing O_2_^•−^ that subsequently dismutates into H_2_O_2_. The prooxidant function of vitamin C is observed at pharmacological doses (millimolar concentrations). It is currently accepted that the anticancer effect of pharmacological doses of ascorbate is mediated by the generation of high concentrations of extracellular H_2_O_2_ that diffuses through the plasma membrane causing DNA damage and triggering proapoptotic signaling pathways which will ultimately lead to cell death [146, 147]. A recent study investigated the synergistic action of pharmacological doses of vitamin C in combination with chemotherapeutic drugs used in the clinic for the treatment of ovarian cancer. These results showed that vitamin C significantly increased the efficacy of carboplatin and/or paclitaxel using a tumorigenic mouse model, where ovarian cancer cells were intraperitoneally injected in the mice [147]. A small pilot phase 1/2a clinical trial was also conducted in patients newly diagnosed with stage III/IV ovarian cancer [147]. Reduction of chemotherapy-associated toxicity was observed in patients receiving intravenous vitamin C in addition to carboplatin and paclitaxel treatment. A tendency for enhanced overall survival and longer time to relapse in patients receiving vitamin C in combination with carboplatin and paclitaxel compared to patients receiving only carboplatin and paclitaxel was observed; however these results were not statistically significant. A larger clinical trial might be useful to further clarify these results. The use of ascorbate in cancer therapy is still a matter of debate as preclinical studies have shown a large variation in ascorbate sensitivity even within cancers of the same type. This may be the result of intrinsic differences between cancer cells from diverse origins (e.g., antioxidant defenses) or the tumor microenvironment in the case of organ-specific cancers, which are presently poorly understood. The fact that vitamin C can function as an antioxidant (at low doses) or a prooxidant (at high doses) is most likely at the basis of these contradictory results and dosage as well as other factors (e.g., intrinsic antioxidant defenses, expression of sodium-dependent vitamin C transporters, activation of signaling pathway(s), etc.) should be taken into account when considering the use of this compound in combination with ROS producing chemotherapeutics.

### 3.9. Others

#### 3.9.1. Annexin A2

Annexin A2 belongs to the family of annexins, which are commonly described as calcium-dependent phospholipid-binding proteins [9]. Annexin A2 is a multifunctional protein that has been shown to be overexpressed in a large number of cancers (e.g., breast, liver, gastric, pancreatic, lung, gliomas, colorectal, and ovarian) and to be positively associated with metastasis and resistance to chemotherapy [9, 148–151]. It was demonstrated that annexin A2 is unique among the vertebrate annexins in that it possesses an N-terminal reactive cysteine residue, Cys-8, which is reversibly oxidized by H_2_O_2_ inactivating this ROS molecule and generating H_2_O and O_2_. Oxidized annexin A2 is then recycled/reduced by the Trx system. Thus one molecule of annexin A2 has the ability to degrade several molecules of H_2_O_2_ [9, 58]. The role of the antioxidant function of annexin A2 in tumorigenesis was investigated using a mouse animal model. This study showed that the growth of tumors derived from annexin A2 depleted cancer cells was significantly impaired compared to tumors derived from control cancer cells (expressing normal levels of annexin A2). However, the growth impairment of the annexin A2-depleted tumors was rescued by the administration of the antioxidant compound, N-acetyl cysteine (NAC), in the mice during tumor formation [9, 58]. These results indicate that annexin A2 REDOX regulatory function plays a significant role in promoting tumor growth. It was also demonstrated that annexin A2 depleted cancer cells were significantly more sensitive to death induced by the ROS generating chemotherapeutic agents, etoposide, doxorubicin, and tamoxifen [58], elucidating for the first time a molecular mechanism by which annexin A2 provides resistance to chemotherapy, by functioning as an antioxidant protein. More recently it has been shown that a fraction of cellular annexin A2 translocates into the nucleus in response to different oxidative stress stimuli, including X-ray radiation, Gamma-radiation, UV radiation, etoposide, chromium VI, and H_2_O_2_ [9, 152, 153]. Nuclear annexin A2 was shown to protect cellular DNA from oxidative damage [153].

#### 3.9.2. DJ-1

DJ-1 is a multifunctional protein that has been shown to also have antioxidant functions. DJ-1 contains three reactive cysteine residues, namely, Cys46, Cys53, and Cys106, being Cys 106 the most susceptible to oxidation. In fact, Cys 106 is essential for DJ-1 cytoprotective function against oxidative stress and mutation of this cysteine residue abolishes all functions of DJ-1 [154–158]. The antioxidant functions attributed to DJ-1 include the upregulation of intracellular glutathione synthesis via increasing glutamate cysteine ligase enzyme [159]; inhibition of oxidative stress induced apoptosis via direct binding of its Cys 106 residue to ASK1 [156, 160]; stabilization of NRF2 transcription factor that regulates the expression of many antioxidant genes (as described above) by preventing the binding of NRF2 to its inhibitor KEAP1 [161]. A recent study using cardiac cells showed that DJ-1 was able to inhibit ischemia/reperfusion-induced ROS generation, via upregulation of antioxidant enzymes [162]. These results suggest that DJ-1 might also trigger a similar antioxidant response in hypoxic cancer cells. However this has not yet been investigated.

DJ-1 has been shown to be upregulated in many human cancer types (e.g., lung, prostate, endometrial, and bladder), which correlated with cancer cell proliferation, tumor survival, and chemoresistance [59, 163–165].

## 4. ROS Inducing Chemotherapy

Typically, cancer cells exhibit higher levels of endogenous ROS compared to normal cells. For this reason a commonly used strategy for killing cancer cells consists of using ionizing radiation and/or chemotherapeutic drugs that induce the generation of these oxidants to levels that are capable of triggering apoptosis once ROS levels reach or exceed a certain threshold within the cell. The rationale for this approach relies on the fact that since cancer cells already have high levels of endogenous ROS prior to treatment they should reach this apoptotic threshold faster/easier compared to normal cells [166, 167].

Accordingly, oxidative stress has been recognized as a tumor specific target for the design of chemotherapeutic agents. A large number of chemotherapeutic drugs capable of inducing oxidative stress, by interfering with various antioxidant/redox regulatory proteins and/or ROS inducing pathways, are currently being used in the clinic (Table 1) [168].

The chemotherapeutic agent carmustine interferes with the GSH antioxidant system by inhibiting glutathione reductase activity. GSH is an abundant cellular antioxidant that plays a key role in cellular REDOX homeostasis as already described in this review. Inhibition of glutathione reductase leads to the build-up of oxidized glutathione (GSSG) that can no longer exert its antioxidant function; as a consequence a significant accumulation of ROS within the cell is observed, driving caspase-3 activation and apoptosis [166, 169, 170].

Other chemotherapeutic drugs function through ROS-independent as well as ROS-inducing mechanisms. This is the case for the estrogen receptor inhibitor, tamoxifen, and for 2-dimethylamino-4,5,6,7-tetrabromo-1H-benzimidazole (DMAT) that inhibit the catalytic activity of protein cyclin kinase 2 (CK2). Under normal conditions, CK2 phosphorylates the apoptosis repressor with caspase recruitment domain (ARC) and Bid (proapoptotic member of the Bcl-2 family) proteins. Phosphorylated ARC localizes to the mitochondria, inhibiting caspase 8, while Bid phosphorylation by CK2 protects it from being cleaved by caspase 8. In conjunction phosphorylation of ARC and Bid by CK2 contributes to cell survival via inhibition of apoptosis. However, when CK2 is inhibited by tamoxifen or DMAT, it is not able to phosphorylate neither ARC nor Bid proteins. This will lead to caspase 8 activation, which will cleave Bid promoting its translocation to the mitochondrial membrane with the subsequent release of cytochrome C and activation of additional caspases, ultimately triggering apoptosis [171–173]. Disruption of the mitochondrial membrane by Bid will also lead to the release of ROS into the cytosol. CK2 inhibition also mediates NOX activation and the subsequent generation of ROS (O_2_^•−^ and H_2_O_2_). This occurs because CK2 can phosphorylate the cytosolic subunit of NOX, p47phox, negatively regulating NOX activity. Inhibition of CK2 by tamoxifen or DMAT will thus enhance NOX activity and induce the elevation of intracellular ROS which will also contribute to cell death induced by these chemotherapeutic agents [174–176].

Paclitaxel (also known as taxol) is another drug that is able to enhance NOX activity. This chemotherapeutic is a microtubule stabilizing agent that interferes with the normal breakdown of spindle microtubules during cell division, inhibiting mitosis and inducing apoptosis [177]. Paclitaxel has been shown to induce the translocation of Rac1 (regulatory subunit of NOX) from the cytosol to the plasma membrane. The increase of Rac 1 in the plasma membrane promoted the activation of NOX and led to the production of O_2_^•−^, which was subsequently converted to H_2_O_2_ either spontaneously or by SOD [178]. This study showed that paclitaxel displayed a significant cytotoxic bystander effect in neighboring cancer cells [178], suggesting that this effect might play a substantial role in the antitumor activity of this drug. It is noteworthy that paclitaxel has limited ability to reach cancer cells that are distant from the vasculature. The fact that H_2_O_2_ can diffuse reasonably far from its site of production and can use aquaporins to enter the cells suggest that this mechanism might contribute significantly to paclitaxel induced cancer cytotoxicity.

The chemotherapeutic drugs actinomycin D and mitomycin C also activate the mitochondria-dependent apoptotic pathway. This occurs via inactivation of the antiapoptotic protein, Bcl-2. Bcl-2 forms heterodimers with the proapoptotic Bcl-2 family members: Bax, Bak, Bid, BIM, PUMA, and BAD sequestering these proteins and inhibiting apoptosis. Inactivation of Bcl-2 by actinomycin D or mitomycin C will elicit the release of the proapoptotic Bcl-2 family members causing mitochondrial membrane potential collapse and cytochrome C release from the mitochondria into the cytosol, subsequently increasing intracellular ROS levels. In the cytosol, cytochrome C induces apoptosis through the activation (via cleavage) of caspase-3 and caspase-9. Excessive ROS generation also contributes to cell death induced by these chemotherapeutic drugs [179–181].

A wide variety of DNA damaging (genotoxic) chemotherapeutic agents have been shown to induce cancer cell death in part through the increase of intracellular ROS levels. This is the case of busulfan, an alkylating agent that causes significant DNA damage by promoting the crosslinking between DNA molecules and also between DNA and proteins. Since these crosslinks can potentially originate DNA strand breaks it was hypothesized that busulfan most likely activates the DNA-damage response p53 signaling pathway to induce senescence or apoptosis. Interestingly, a p53 independent pathway has been reported for busulfan induced senescence. It was demonstrated that busulfan is metabolized through conjugation with GSH. This reaction is catalyzed by GST. This leads to depletion of intracellular levels of GSH, followed by a continuous increase in ROS production via NOX activation, which in turn activate the ERK and p38 MAPK signaling pathways, inducing cell senescence [182–184].

Bleomycin (BLM) is a chelator and a DNA damaging agent that is known to produce single- and double-strand breaks in the DNA as well as the release of free bases. BLM binds to DNA via its bithiazole and N-terminal moieties and interacts with Fe(II) via its ferrous binding site, forming an Fe(II)-BLM-DNA complex. BLM induces an increase in intracellular levels of ROS due to oxidation of the Fe(II)-BLM-DNA complex by O_2_ that leads to the generation of O_2_^•−^ and ^•^OH [185–187].

Other genotoxic chemotherapeutics, such as doxorubicin and 5-Fluorouracil, trigger a p53-dependent proapoptotic pathway in order to kill cancer cells. The tumor suppressor p53 is involved in the maintenance of DNA integrity and under normal conditions it is maintained at low levels by the E3 ubiquitin ligase MDM2. Doxorubicin and 5-fluorouracil induce the stabilization of p53 and the subsequent transcription of p53 regulated genes including cytochrome oxidase 2, a component of the cellular respiratory chain that has been shown to generate ROS [188]. Another molecular mechanism by which 5-Fluorouracil induces the generation of ROS is by p53-dependent transcription of the ROS modulator 1 (ROMO1) gene. ROMO 1 protein localizes at the mitochondrial membrane and induces mitochondrial ROS generation [188, 189].

Doxorubicin also induces cancer cell apoptosis in a p53-independent way. Both doxorubicin and etoposide genotoxic chemotherapeutics promote the accumulation and nuclear translocation of the forkhead transcription factor, FOXO3, which activates the transcription of the proapoptotic genes Noxa and BIM. This promotes the collapse of the mitochondrial membrane with the subsequent release of cytochrome C and ROS into the cytosol ultimately inducing apoptosis [190–192]. Another mechanism by which doxorubicin produces ROS involves the addition of one-electron to the quinone moiety of doxorubicin resulting in the formation of a semiquinone that quickly regenerates into a quinone by reducing O_2_ to O_2_^•−^ and H_2_O_2_. The one-electron redox cycling of doxorubicin is accompanied by a release of iron from intracellular stores; binding of doxorubicin with iron results in formation of 3 : 1 drug-iron complexes that convert O_2_^•−^ and H_2_O_2_ into more potent ^•^OH [193].

Doxorubicin is known to produce serious side effects, the most dangerous of which being cardiomyopathy which has been closely associated with abnormalities in mitochondrial functions including defects in the respiratory chain/oxidative phosphorylation system, decreased ATP production, mitochondrial DNA damage, modulation of mitochondrial sirtuin activity, and ROS generation [193–196]. For this reason it is important to take into account that using ROS to kill cancer cells might also have serious consequences for the patient that need to be taken into serious consideration before administration of these chemotherapeutic compounds.

The chemotherapeutic drug, cisplatin, is an alkylating agent that causes DNA crosslinking ultimately leading to p53-dependent apoptosis. It has been shown that DNA damage induced by cisplatin triggers a p53-dependent upregulation of ROS that activate p38 and JNK signaling pathways inducing apoptosis in a wide variety of cancer cell lines [188, 197].

The chemotherapeutic gemcitabine (2′-2′ difluorodeoxy cytidine, dFdC) is a prodrug that is phosphorylated by deoxycytidine kinase within the cell to form the active compounds gemcitabine diphosphate (dFdCDP) and gemcitabine triphosphate (dFdCTP). The active forms of gemcitabine incorporate into the DNA, causing cell cycle arrest and subsequent apoptosis [198]. It has also been shown that gemcitabine induces ROS as an additional anticancer mechanism [199]. Interestingly a report has shown that gemcitabine induced ROS activate the proproliferative and prosurvival MAPKS, ERK1/2, and AKT. The activation of these pathways leads to the enhanced nuclear accumulation of NF-kB and HIF-1*α* transcription factors which induce the transcription and subsequent expression of CXCR4 protein [198, 200]. These authors also demonstrated that increased expression of CXCR4 enhanced cancer cell invasion and migration to CXCL12 chemokine rich environments, namely, the stroma and blood vessels, promoting in this way chemoresistance due to escape from the tumor site [198]. For this reason, ROS concentrations within the cell should be considered during chemotherapy treatment since survival and metastatic mechanisms can be induced. It is crucial that ROS concentrations are increased until a threshold is reached where the cellular balance is tipped to activate apoptotic pathway(s) to stop tumor progression [176, 200]. Although high levels of ROS induced by chemotherapeutics can have dangerous side effects (as seen for doxorubicin), low dosages can also promote tumor growth instead of death. It becomes clear that finding the optimal dosage for the use of these chemotherapeutic agents is crucial for an effective cancer therapy.

All of the chemotherapeutics described above (summarized in Table 1) generate ROS that will contribute to tumor cell death. This is because excessive ROS can induce significant DNA damage, protein oxidation, organelles, and/or membranes oxidation tipping the cellular balance away from the proliferative advantages of low/moderate ROS levels to drive apoptosis in the presence of high ROS levels [201].

## 5. Conclusions and Future Directions

Increasing evidence has established that the cellular antioxidant systems play a key role not only in regulating (normal) cellular redox homeostasis, but also in protecting cancer cells from the increasing oxidative stress that they are subjected to during tumor progression. A large number of antioxidant proteins have been shown to be upregulated in cancer and to promote resistance to chemotherapy. These include not only proteins of the GSH and Trx systems that play a major role in recycling/reducing REDOX sensitive proteins, whose function is regulated by oxidation/reduction of key reactive cysteine residues, but also ROS scavenging proteins and transcription factors that induce the cellular antioxidant response. Novel antioxidant proteins, such as annexin A2 and DJ-1, have also been identified more recently that contribute significantly to tumorigenesis. In summary, since it was established that tumors are typically under substantial oxidative stress and as such have to adapt to survive in this extreme environment; increasing interest has been drawn to identifying the antioxidant/redox regulatory proteins involved in the tumor REDOX adaptation and in understanding the molecular mechanisms by which these proteins promote tumor progression and resistance to chemotherapy. Taking into account that many chemotherapeutic drugs currently in use in the clinic rely to varying degrees on ROS overload to kill the cancer cells, the tumor REDOX adaptation presents a major obstacle for the efficacy of these therapies. One approach to overcome this problem could be to deplete the cancer cell from REDOX regulatory potential through the downregulation of antioxidant protein(s) and peptides that have been shown to play crucial roles for tumor survival and growth in combination with chemotherapeutics that induce ROS.

## Figures and Tables

**Table 1:** Chemotherapeutic drugs commonly used in the clinic capable of inducing ROS.

Drug name	Tumor type	Mechanism of action for ROS induction	References
Actinomycin D	Sarcomas; Wilms' tumor; testicular; melanoma; neuroblastoma; germ cell; retinoblastoma; choriocarcinoma	Inhibition of Bcl-2	[179]

Bleomycin	Melanoma; Hodgkin's and non-Hodgkin's lymphomas; testicular; head and neck; cervical; malignant pleural effusions	Formation of Fe(II)-bleomycin-DNA complex that is oxidized by O_2_	[185, 187]

Busulfan	Chronic myeloid leukemia	GSH depletion and NOX activation	[182, 183]

Carmustine	Brain; Hodgkin's and non-Hodgkin's lymphoma; melanoma; myeloma	GSH depletion via inhibition of GR	[169]

Cisplatin	Ovarian; colon; testicular; germ cell; bladder; lung; head and neck	Increased expression of p47^phox^ subunit of NOX	[17, 188]

DMAT	Prostate	Inhibition of CK2 activity	[173]

Doxorubicin	Hodgkin's and non-Hodgkin's lymphoma; leukemia; breast; gastric; neuroblastoma; ovarian; lung; soft tissue and bone sarcomas; thyroid; bladder	p53-dependent transcription of cytochrome oxidase 2; FOXO3-dependent transcription of Noxa and BIM; quinone metabolism	[190]

Etoposide	Lymphomas; leukemias; neuroblastoma; breast; lung; testicular; gastric	FOXO3 dependent transcription of Noxa and BIM	[188, 190]

5-Fluorouracil	Gastric; colon; gynecological; breast; head and neck; lung; skin	p53-dependent transcription of ROMO 1	[188, 189]

Gemcitabine	Pancreatic; lung; in combination with other drugs: breast, bladder, and ovarian	Activation of AKT and ERK 1/2 which leads to upregulation of CXCR4	[198]

Mitomycin C	Colon; breast; head and neck; bladder; cervical; gastric; pancreatic; liver	Inhibition of Bcl-2	[180, 202]

Paclitaxel	Ovarian; breast; non-small cell lung carcinoma; Kaposi's sarcoma	Activation of Rac1 subunit of NOX; disruption of the mitochondrial membrane	[177, 178]

Tamoxifen	Breast	Inhibition of CK2 activity	[175]
